# First rebbachisaurid sauropod dinosaur from Asia

**DOI:** 10.1371/journal.pone.0246620

**Published:** 2021-02-24

**Authors:** Alexander Averianov, Hans-Dieter Sues

**Affiliations:** 1 Department of Theriology, Zoological Institute, Russian Academy of Sciences, St. Petersburg, Russia; 2 Department of Paleobiology, National Museum of Natural History, Smithsonian Institution, Washington, D.C., United States of America; Perot Museum of Nature and Science, UNITED STATES

## Abstract

*Dzharatitanis kingi* gen. et sp. nov. is based on an isolated anterior caudal vertebra (USNM 538127) from the Upper Cretaceous (Turonian) Bissekty Formation at Dzharakuduk, Uzbekistan. Phylogenetic analysis places the new taxon within the diplodocoid clade Rebbachisauridae. This is the first rebbachisaurid reported from Asia and one of the youngest rebbachisaurids in the known fossil record. The caudal is characterized by a slightly opisthocoelous centrum, ‘wing-like’ transverse processes with large but shallow PRCDF and POCDF, and the absence of a hyposphenal ridge and of TPRL and TPOL. The neural spine has high SPRL, SPDL, SPOL, and POSL and is pneumatized. The apex of neural spine is transversely expanded and bears triangular lateral processes. The new taxon shares with *Demandasaurus* and the Wessex rebbachisaurid a high SPDL on the lateral side of the neural spine, separated from SPRL and SPOL. This possibly suggests derivation of *Dzharatitanis* from European rebbachisaurids. This is the second sauropod group identified in the assemblage of non-avian dinosaurs from the Bissekty Formation, in addition to a previously identified indeterminate titanosaurian.

## Introduction

Exposures of the Upper Cretaceous Bissekty Formation along the escarpment at Dzharakuduk in the central Kyzylkum Desert of Uzbekistan have yielded a vast number of mostly dissociated but often exquisitely preserved skeletal remains of vertebrates, including those representing a great diversity of non-avian dinosaurs [1]. Among this material are isolated bones and teeth of sauropods, which formed the subject of previous studies by Sues et al. [2] and Averianov and Sues [3, 4]. This occurrence is of great interest because relatively few records of sauropod remains are known to date from stratigraphically well-constrained strata of the Upper Cretaceous in Central Asia [4]. The Bissekty Formation is an up to 80 m thick succession of medium-grained, poorly lithified, cross-bedded fluvial sandstones and clast-supported, well-cemented intraformational conglomerates [5]. The chronostratigraphic position of the unit has been bracketed using invertebrate fossils from marine units overlying and underlying the Bissekty Formation at Dzharakuduk as well as comparisons with other Late Cretaceous vertebrate assemblages known from Central Asia [6]. The unit is considered middle to late Turonian in age.

Sues et al. [2] and Averianov and Sues [4] interpreted the sauropod remains from the Bissekty Formation as referable to a non-lithostrotian titanosaur. The braincase CCMGE 628/12457 was first interpreted as belonging to a derived titanosaur [2, 7–9], but, with a larger sample of endocranial casts for the titanosaurs available now, the phylogenetic position of this taxon is probably more basal [10]. All sauropod fossils from the Bissekty Formation are disassociated but usually well-preserved bones and teeth. The nature of the depositional environment makes it unlikely that even partial articulated sets of skeletal elements will ever be recovered. However, the anterior caudal vertebra from the Bissekty Formation discussed in this paper (USNM 538127) is clearly distinguished by its intricate lamination and extensive pneumatization of the neural arch. It does not compare closely to the proximal caudals of the numerous titanosaurian taxa described to date. However, it shares important features with diplodocoid sauropods and, more specifically, rebbachisaurids. The Rebbachisauridae were previously known only from the mid-Cretaceous of Africa, Europe, and South America. In this paper, we describe the anterior caudal USNM 538127 as the first record of a rebbachisaurid from the Late Cretaceous of Asia. It also represents one of the geologically youngest known records of Rebbachisauridae.

The nomenclature of the vertebral fossae and laminae follows Wilson [11, 12] and Wilson et al. [13]. As all rebbachisaurid genera discussed in this paper are monotypic we use only the generic names. USNM 538127 was scanned using Artec Spider and its three-dimensional model was produced in Artec Studio 13 and Geomagic Studio.

### Institutional abbreviations

CCMGE, Chernyshev’s Central Museum of Geological Exploration, Saint Petersburg, Russia; USNM, Department of Paleobiology, National Museum of Natural History, Smithsonian Institution, Washington, D.C., U.S.A.; ZIN PH, Paleoherpetological Collection, Zoological Institute, Russian Academy of Sciences, Saint Petersburg, Russia.

### Anatomical abbreviations

CPOL, centropostzygapophyseal lamina; CPRL, centroprezygapophyseal lamina; POCDF, postzygapophyseal centrodiapophyseal fossa; PODL, postzygodiapophyseal lamina; POSDF, postzygapophyseal spinodiapophyseal fossa; POSL, postspinal lamina; PRCDF, prezygapophyseal centrodiapophyseal fossa; PRDL, prezygodiapophyseal lamina; PRSDF, prezygapophyseal spinodiapophyseal fossa; PRSL, prespinal lamina; SDF, spinodiapophyseal fossa; SPDL, spinodiapophyseal lamina; SPOF, spinopostzygapophyseal fossa; SPOL, spinopostzygapophyseal lamina; SPDL, spinodiapophyseal lamina; SPRF, spinoprezygapophyseal fossa; SPRL, spinoprezygapophyseal lamina; TPOL, interpostzygapophyseal lamina; TPRL, interprezygapophyseal lamina.

### Nomenclatural acts

The electronic edition of this article conforms to the requirements of the amended International Code of Zoological Nomenclature, and hence the new names contained herein are available under that Code from the electronic edition of this article. This published work and the nomenclatural acts it contains have been registered in ZooBank, the online registration system for the ICZN. The ZooBank LSIDs (Life Science Identifiers) can be resolved and the associated information viewed through any standard web browser by appending the LSID to the prefix "http://zoobank.org/". The LSID for this publication is: urn:lsid:zoobank.org:pub:9BF5E3F9-17A9-4159-A063-D83394AD6DBC. The electronic edition of this work was published in a journal with an ISSN, and has been archived and is available from the following digital repositories: PubMed Central and LOCKSS.

### Systematic paleontology

**Sauropoda** Marsh, 1878 [14]
**Neosauropoda** Bonaparte, 1986 [15]
**Diplodocoidea** Marsh, 1884 [16]
**Rebbachisauridae** Bonaparte, 1997 [17]
Genus ***Dzharatitanis*** gen. nov.
urn:lsid:zoobank.org:act:1EEBE657-A1F2-4165-9E83-43479ED5CE6B

### Type species

*Dzharatitanis kingi* sp. nov.

### Diagnosis

Differs from *Limaysaurus* and *Tataouinea* by convex anterior centrum articular surface. Differs from *Lavocatisaurus* and *Limaysaurus* by absence of pleurocoel on centrum. Differs from *Comahuesaurus* by ‘wing-like’ transverse process. Differs from *Amazonsaurus* by dorsally directed ventral surface of transverse process. Differs from *Cathartesaura*, *Comahuesaurus*, *Demandasaurus*, *Itapeusaurus*, and *Tataouinea* by shallow PRCDF. Differs from *Cathartesaura*, *Itapeusaurus*, *Katepensaurus*, and *Tataouinea* by absence of TPRL. Differs from *Comahuesaurus*, *Demandasaurus*, and *Nigersaurus* by absence of ventral contact between prezygapophyses. Differs from *Comahuesaurus*, *Demandasaurus*, *Itapeusaurus*, *Limaysaurus*, *Nigersaurus*, and *Tataouinea* by absence of ventral contact between postzygapophyses. Differs from *Demandasaurus* and *Tataouinea* by absence of hyposphenal ridge. Differs from *Amazonsaurus*, *Cathartesaura*, *Katepensaurus*, *Limaysaurus*, *Nigersaurus*, and *Tataouinea* by absence of SPRL and SPOL contact. Differs from *Amazonsaurus*, *Cathartesaura*, *Katepensaurus*, *Limaysaurus*, *Nigersaurus*, *Tataouinea*, and *Rebbachisaurus* by large SPDL on lateral side of neural spine separate from SPRL and SPOL. Differs from *Amazonsaurus*, *Cathartesaura*, *Comahuesaurus*, *Itapeusaurus*, *Lavocatisaurus*, and *Limaysaurus* by presence of lateral process of neural spine. Differs from *Rebbachisaurus* by proximodistally shorter and anteroposteriorly wider neural spine, which is convex anteriorly in lateral view, and by much wider PRSL.

### Referred species

Type species only.

### Occurrence

Central Asia; Late Cretaceous (Turonian).

### Etymology

From the Dzharakuduk locality in Uzbekistan and Greek τιτάν (titan), a pre-Olympian god in ancient Greek mythology. The generic name is in the feminine gender.

***Dzharatitanis kingi* sp. nov.** urn:lsid:zoobank.org:act:E3DB1711-8BD1-4834-8268-5A11004C0463 2015 Titanosauria indet.: figure 7 in [2].

### Holotype

USNM 538127, nearly complete anterior caudal vertebra. Found by David J. Ward and Hans-Dieter Sues during the URBAC (Uzbekistan/Russian/British/American/Canadian) joint paleontological expedition working in Uzbekistan in 1997.

### Referred specimens

Holotype only.

### Type locality and horizon

Dzharakuduk, 32 km SW of Mynbulak, Navoi Viloyat, Uzbekistan. The Bissekty Formation, exposed along the Dzharakuduk escarpment, extends from approximately 42°06’22.60’’ N and 62°37’09.00’’ E to 42°05’44.22’’ N and 62°41’06.49’’ E. Age: Late Cretaceous (Turonian). For additional geological details see Redman and Leighton [5].

### Diagnosis

As for the genus.

### Etymology

In memory of our colleague and friend Dr. Christopher King (1943–2015) who did much work on the geology of Cretaceous strata in Central Asia.

### Remarks

USNM 538127 is likely the first caudal vertebra because of its slightly opisthocoelous centrum and the absence of chevron facets. First caudals with opisthocoelous centra are known for several rebbachisaurids (see Comparison).

### Description

**Fig 1:**
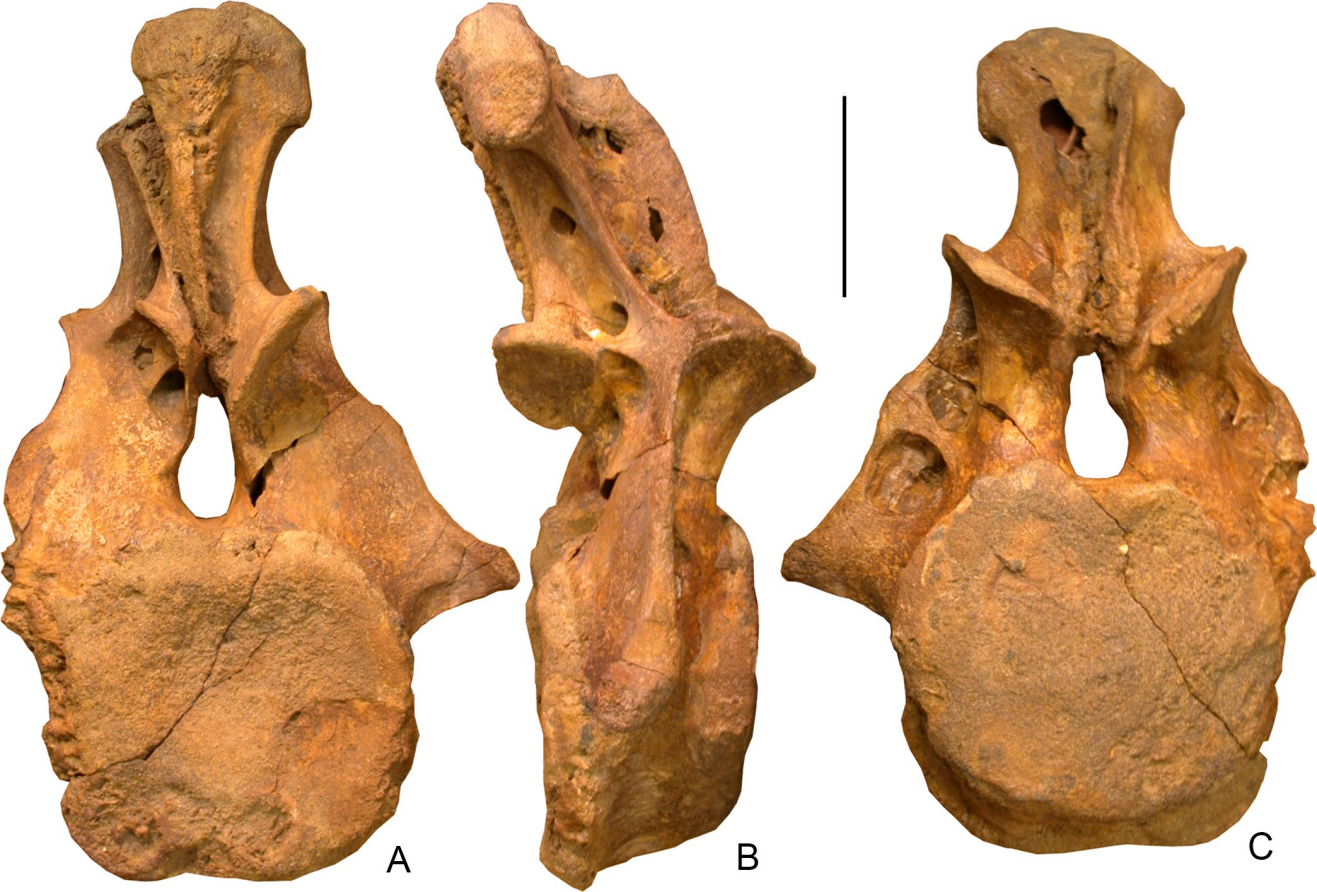
*Dzharatitanis kingi*, USNM 538133 (holotype), anterior caudal vertebra in posterior **(A)**, right lateral **(B)**, and anterior **(C)** views. Scale bar = 10 cm.

**Fig 2:**
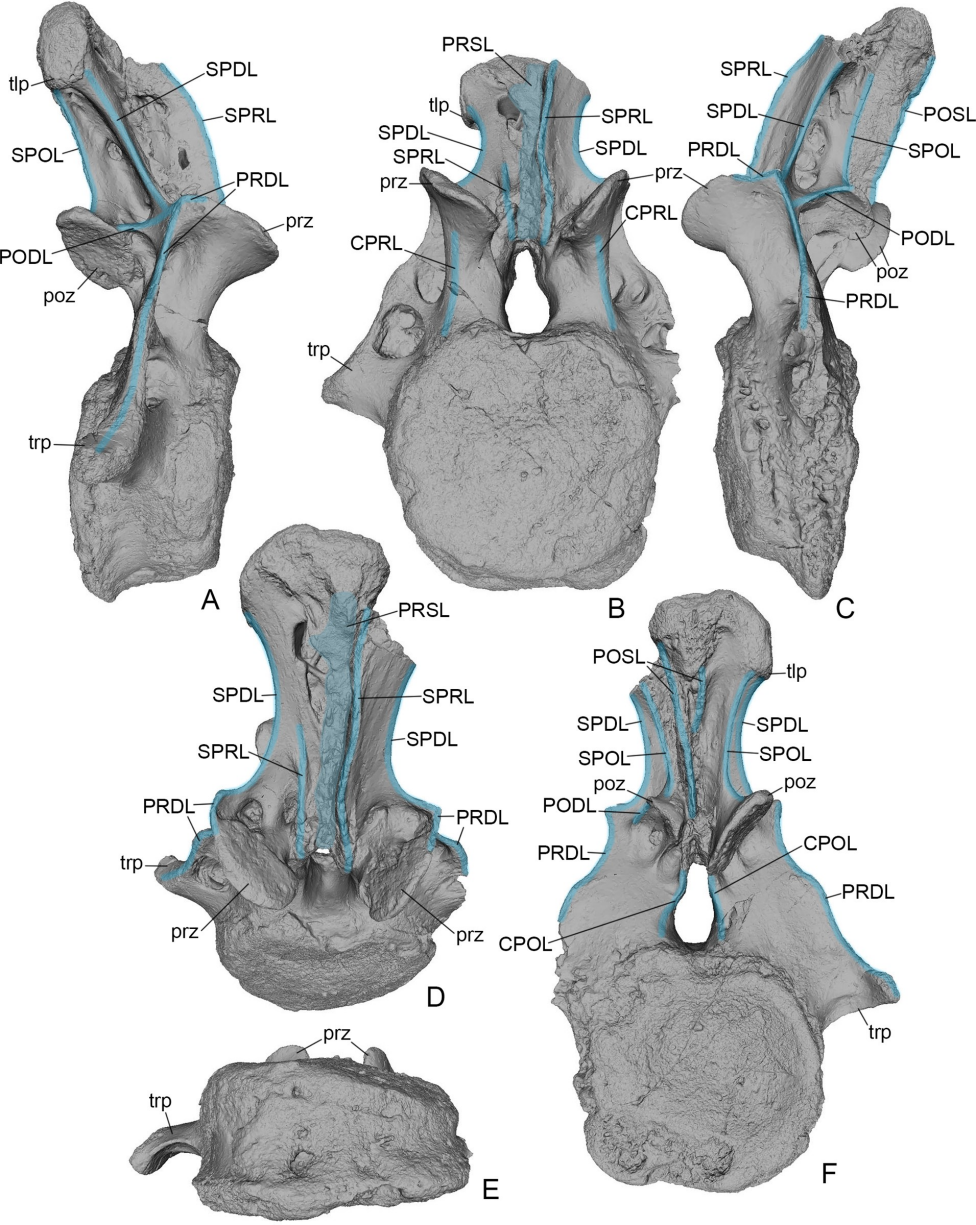
*Dzharatitanis kingi*, USNM 538133 (holotype), digital renderings of the anterior caudal vertebra in right lateral **(A)**, anterior **(B)**, left lateral **(C)**, anterodorsal **(D)**, ventral **(E)**, and posterior **(F)** views. The laminae are highlighted in blue. Abbreviations: CPOL, centropostzygapophyseal lamina; CPRL, centroprezygapophyseal laminae; PODL, postzygodiapophyseal lamina; POSL, postspinal lamina; poz, postzygapophysis; PRDL, prezygodiapophyseal lamina; PRSL, prespinal lamina; prz, prezygapophysis; SPDL, spinodiapophyseal lamina; SPOL, spinopostzygapophyseal lamina; SPRL, spinoprezygapophyseal lamina; tlp, triangular lateral process; trp, transverse process. Not to scale.

**Fig 3:**
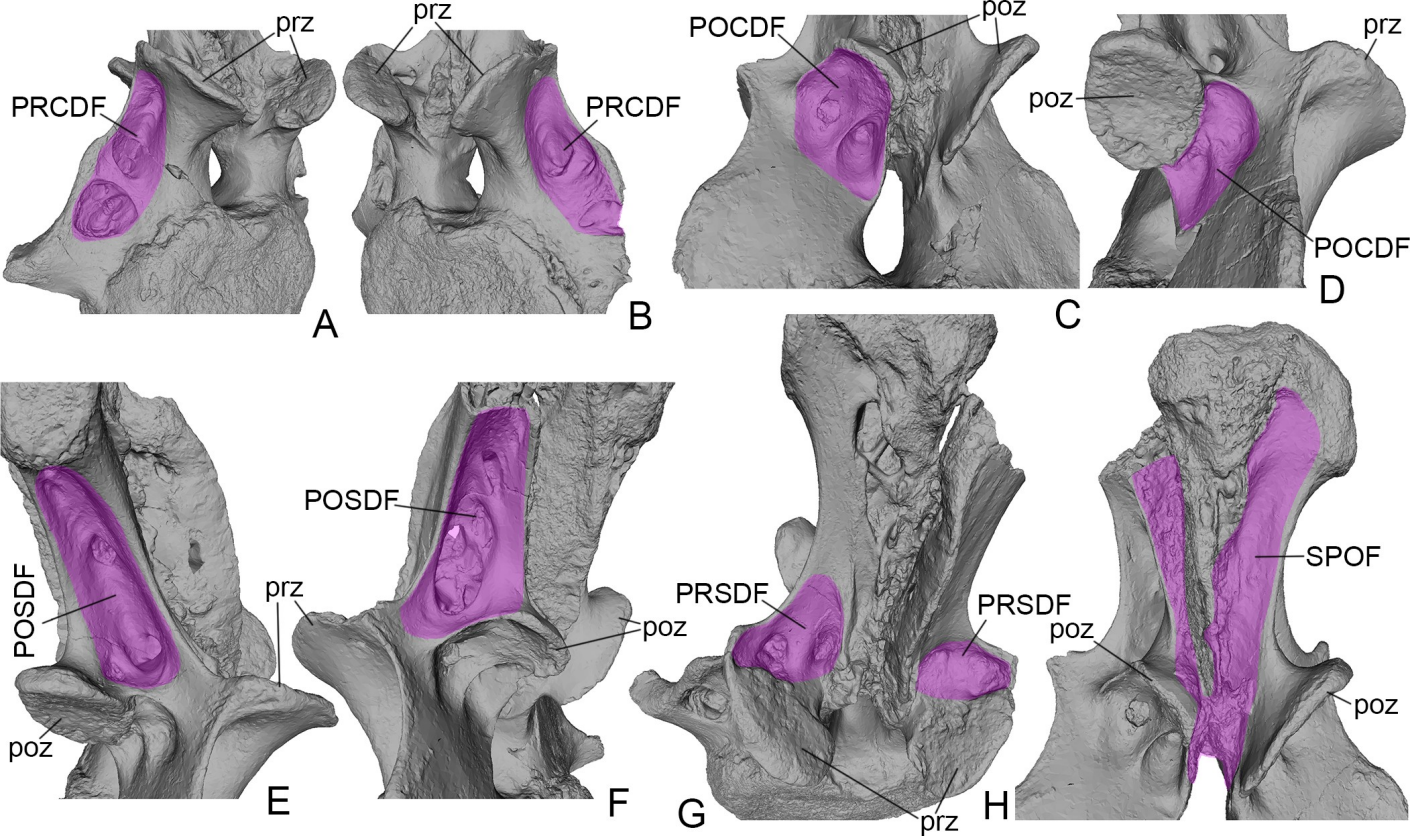
*Dzharatitanis kingi*, USNM 538133 (holotype), digital renderings of anterior caudal vertebra in right anterolateral **(A)**, left anterolateral **(B)**, left posterolateral **(C)**, right ventrolateral **(D)**, right lateral **(E)**, left lateral **(F)**, anterodorsal **(G)**, and posterior **(H)** views. The fossae are highlighted in purple. Abbreviations: POCDF, postzygapophyseal centrodiapophyseal fossa; POSDF, postzygapophyseal spinodiapophyseal fossa; poz, postzygapophysis; PRCDF, prezygapophyseal centrodiapophyseal fossa; PRSDF, prezygapophyseal spinodiapophyseal fossa; prz, prezygapophysis; SPOF, spinopostzygapophyseal fossa. Not to scale.

USNM 538127 is a nearly complete caudal vertebra lacking the left transverse process (Figs 1–3). The centrum is short anteroposteriorly, with the centrum length (9.8 cm) only 55 percent the height of the anterior articular surface of the centrum (17.9 cm). The anterior articular surface of the centrum is placed higher than the posterior one and thus the ventral surface of the centrum extends posteroventrally in lateral view (Figs 1B and 2A and 2C). The centrum is opisthocoelous, with the anterior articular surface being slightly convex and the posterior articular surface more deeply concave. The anterior articular surface is slightly higher than wide (height: 17.9 cm; width: 16.4 cm) whereas the opposite obtains for the posterior one (height: 16.9 cm; width: 18.1 cm). Both surfaces are more or less round in outline, with a somewhat flattened ventral margin and with a concavity along the dorsal margin at the ventral floor of the neural canal. The lateral surface of the centrum is convex dorsoventrally and concave anteroposteriorly. The ventral surface is flat to slightly concave, without longitudinal grooving or ridges. There is a large vascular foramen on the right side and a much smaller opening on the left side (Fig 2E). There are no chevron facets.

The right transverse process is completely preserved. Its ventral margin is slightly dorsal to the mid-height of the centrum. The proximodistal length of the transverse process is about one third of the width of the anterior articular surface of the centrum. The distal part of the transverse process curves posterolaterally. The dorsal margin of the transverse process is formed by a distinct lamina that connects with the prezygapophysis and thus can be interpreted as the PRDL (Fig 2A and 2C). This lamina connects at the level of the zygapophyses with the SPDL and PODL (Fig 2A and 2C). The PRDL and SPDL enclose an angle of ~135°. The anterior surface of the transverse process bears two deep depressions (PRCDF), which are separated by a transverse bony bar extending into a lateral hump-like projection on the PRDL (Fig 3A and 3B). The ventral depression is larger than the dorsal one on the left transverse process but is smaller on the right transverse process. The ventral depression is round in outline and occupies the dorsal half of the space between the transverse bony bar and the ventral margin of the transverse process. Its deepest point is near its ventral margin. The ventral depression is subdivided by two low oblique bony crests. The dorsal depression is shallower and occupies the entire space between the PRDL, the transverse bony bar, and the prezygapophysis. It becomes deeper ventrally, with the deepest part adjacent to the transverse bony bar. The posterior side of the transverse process is flat except for a shallow groove along the ventral margin of the process.

The neural arch occupies almost the entire length of the centrum. The narrowest point of the pedicle of the neural arch in lateral view is midway between the centrum and zygapophyses. The neural canal is relatively high, about half the height of the anterior articular surface of the centrum, and pear-shaped in anterior and posterior views, with the dorsal part transversely narrower than the ventral part (Fig 2B and 2F). There are no transverse laminae connecting the collateral zygapophyses. The dorsal roof of the neural canal is formed only by the narrow base of the neural spine. This base has a rugose sculpture and continues uninterrupted into the similarly rugose PRSL and POSL anteriorly and posteriorly.

Each of the widely separated prezygapophyses is supported by a pillar-like CPRL (Fig 2B). The right prezygapophysis is somewhat larger than the left. The prezygapophyseal articular surfaces are inclined at an angle of 53° to the horizontal plane. The prezygapophyseal articular surface is teardrop-shaped, round anteromedially and pointed posterolaterally. It is flat to slightly convex. There is no TPRL connecting the prezygapophyses. Instead, prominent ridges extend ventromedially from the ventromedial corner of the prezygapophyseal articular surfaces towards the lateral margin of the neural canal.

The left postzygapophysis has an abraded margin but definitely was much smaller than the right one. The postzygapophyses are well separated but not as widely spaced apart as the prezygapophyses. The postzygapophyseal articular surface is oval and slightly concave. There are two depressions anterolateral to the postzygapophysis (POCDF), which are deeper on the left side of the arch (Fig 3C and 3D). They are bounded by a short PODL dorsolaterally and a distinct CPOL posteromedially (Fig 2F). The depressions are separated by a distinct sharp ridge, which is higher on the left side. The PODL is longer on the left side. The CPOL extends ventrally along the margin of the neural canal and terminates some distance dorsal to its ventral surface (Fig 2F).

The neural spine (maximum anteroposterior length: 9.4 cm; minimum transverse width: 7.5 cm; height: 18.8 cm) is inclined posterodorsally and slightly projects beyond the centrum posteriorly. It is distinctly asymmetrical and has a fracture extending along the entire height of the neural spine on the left side. This break split off about one third of the neural spine, including the left postzygapophysis, left SPOL, left SPDL, and left SPRL. The dorsal portion of this split part of the neural spine is missing and breakage reveals that the neural spine is hollow internally. In anterior or posterior view, the neural spine is constricted above the zygapophyses and is expanded at its apex, forming a mace-like projection. This projection is subdivided into three parts: a medial part with a flattened dorsal surface and two lateral parts of which the left was separated by the fracture and is now missing. The surface of the right lateral part consists of two facets set at an angle to each other, and the medial facet is set an angle to the dorsal surface of the middle part. The lateral facet faces almost laterally and continues ventrally as the triangular lateral process.

The SPRL is highly asymmetrical. It is well-developed on the left side but much reduced on the right side (Fig 2B and 2D). The right SPRL is formed by a short and low ridge at the base of the neural spine. The left SPRL extends between the missing dorsal end of the split part of the neural spine and the ventral end of the spine. It is separated from the prezygapophysis by a deep furrow. The left SPRL is very high, with its highest point in its dorsal half, and hollow internally. The PRSL is a low rugose area occupying the entire space between the collateral SPRLs. The dorsal portion of the neural spine, to the right of the PRSL, has a large opening leading into the interior cavity of the neural spine.

The POSL extends along the entire height of the neural spine (Fig 2F). It considerably expands dorsally, forming a triangular, rugose area that is confluent with the dorsal side of the middle portion of the neural spine. Ventrally, the POSL extends between the postzygapophyses.

The space between the SPRL medially, SPDL laterally, and the prezygapophyseal articular surface ventrally is the PRSDF (Fig 3G). This fossa is deepest in the ventral part, posterior to the prezygapophyseal articular surfaces. Here there are two distinct depressions, the more prominent one on the right side. The lateral depression is larger and deeper. The space between the collateral SPOLs and the POSL is a deep SPOF (Fig 3H). The lateral side of the neural spine, between the SPDL, SPOL, and PODL, is occupied by the POSDF (Fig 3E and 3F). On left side, this fossa contains a prominent dorsal pneumatic foramen and two ventral ones. The ventral openings are separated by a narrow bony strut. On the right side, the ventral half of the fossa contains a large pneumatic foramen and a much smaller foramen dorsal to it.

### Phylogenetic analysis

**Fig 4:**
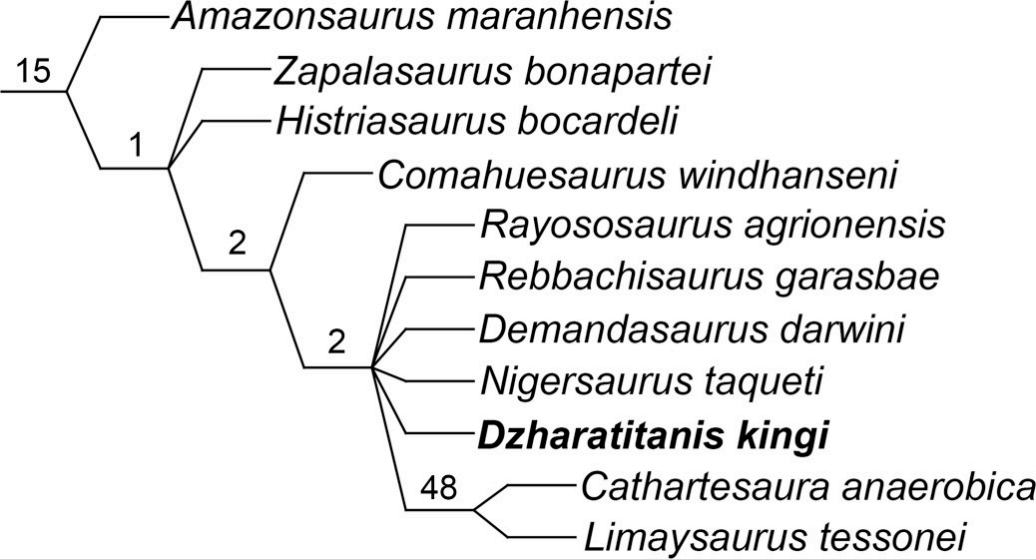
Part of the strict consensus of 1020 trees produced by TNT analysis showing the phylogenetic relationships within Rebbachisauridae and the position of *Dzharatitanis kingi* with GC values at the nodes.

For the phylogenetic analysis we used data matrix of Rauhut et al. [18] as modified by Xu et al. [19]. This matrix consists of 375 characters scored for 74 sauropodomorph taxa. Characters 12, 58, 95, 96, 102, 106, 108, 115, 116, 119, 120; 145, 152, 163, 213, 216, 232–235, 252, 256, 298, 299, and 301 were treated as ordered multistate characters. The scorings for USNM 538127 are listed in S1 File. We also changed some scorings, as discussed in the next section and listed in S1 File. We used TNT vs. 1.5 [20, 21] to analyze the character-taxon matrix. The New Technology Search was applied first, using sectorial searches, ratchet, drift, and tree fusing, with the consensus stabilized 10 times. This search recovered 369 most parsimonious trees (MPTs) with a length of 1146 steps (consistency index = 0.38; retention index = 0.72). These trees were then subjected to the Traditional Search using tree bisection and reconnection (TBR) branch swapping, which produced 1020 MPTs. The consensus tree is shown on Fig 4. We also performed an implied weight analyses (k = 9 and 12) with the same settings. These analyses produced 41 MPTs with 51.35 steps (k = 9) and 45 MPTs with 45.91 steps (k = 12) (consistency index = 0.37; retention index = 0.71). The phylogenetic relationships within Diplodocoidea on the consensus trees are identical to those found in the equal weight analysis. The support for each node in the trees was assessed in TNT using Group present/contradicted (GC) frequency values calculated from 5000 replicates of symmetric resampling, with a probability of weight change (p) of 33% [22].

On the resulting consensus tree, *Dzharatitanis* is placed within Rebbachisauridae in a polytomy with *Rayososaurus*, *Rebbachisaurus*, *Demandasaurus*, *Nigersaurus*, and a clade comprising *Cathartesaura* and *Limaysaurus* (Fig 4). The placement of *Dzharatitanis* within Rebbachisauridae is supported by the absence of the hyposphenal ridge (203(0)). The new taxon belongs to the clade containing Rebbachisauridae more derived than *Amazonsaurus* based on the dorsally directed ventral surface of the transverse process (192(1)). The placement of the new taxon in the clade comprising *Comahuesaurus* and more derived rebbachisaurids is supported by the posterior articular surface of the centrum being markedly more concave than the anterior one (193(4)). It is placed in the clade containing rebbachisaurids more derived than *Comahuesaurus* by the triangular lateral process on neural spine (197(1)) and the ‘wing-like’, not distally tapering transverse process (198(1)).

We performed also the second phylogenetic analysis with the same search protocol but with the matrix expanded to include an additional OTU for the braincase CCMGE 628/12457 from Dzharakuduk [2]. The scorings for this braincase are listed in S1 File. The New Technology Search produced 340 MPTs with tree length of 1146 steps. The Traditional Search based on these trees produced 1272 MPTs. On the strict consensus tree, the position of *Dzharatitanis* is identical to the previous analysis but the braincase CCMGE 628/12457 is recovered within Titanosauriformes, whose interrelationships are largely unresolved. This result indicates the presence of at least two sauropod taxa at Dzharakuduk, an indeterminate titanosaur and the rebbachisaurid *Dzharatitanis kingi*.

### Comparison

In this section we present a morphological comparison between the anterior caudal of *Dzharatitanis kingi* and the anterior caudals of Rebbachisauridae and some other sauropods. The data on geographic and stratigraphic distribution of Rebbachisauridae are shown in Table 1. This list does not include several problematical taxa that cannot be definitively assigned to Rebbachisauridae. The distribution of the diagnostic characters discussed in this section is summarized in Table 2.

**Table 1:** Spatial and temporal distribution of the Rebbachisauridae.

Taxon	Country	Formation	Age	References
*Histriasaurus boscarollii*	Croatia	unnamed	Hauterivian-Barremian	[23–25]
*Demandasaurus darwini*	Spain	Castrillo la Reina	Barremian-Aptian	[26, 27]
*Zapalasaurus bonapartei*	Argentina	La Amaraga	Barremian-Aptian	[28]
*Nigersaurus taqueti*	Niger	Elrhaz	Aptian	[13, 29–31]
*Amazonsaurus maranhensis*	Brazil	Itapecuru	Aptian-Albian	[32]
*Comahuesaurus windhauseni*	Argentina	Lohan Cura	Aptian-Albian	[33, 34]
*Lavocatisaurus agrioensis*	Argentina	Rayoso	Aptian-Albian	[35]
*Tataouinea hannibalis*	Tunisia	Ain el Guettar	Albian	[36, 37]
*Rebbachisaurus garasbae*	Morocco	Kem Kem	Cenomanian	[38–40]
*Itapeusaurus cajapioensis*	Brazil	Alcântara	Cenomanian	[41]
*Rayososaurus agrioensis*	Argentina	Candeleros	Cenomanian	[42, 43]
*Limaysaurus tessonei*	Argentina	Candeleros	Cenomanian	[34, 44]
*Katepensaurus goicoecheai*	Argentina	Bajo Barreal	Cenomanian-Turonian	[36, 45]
*Dzharatitanis kingi*	Uzbekistan	Bissekty	Turonian	This report
*Cathartesaura anaerobica*	Argentina	Huincul	Turonian-Coniacian	[46]

**Table 2:** Distribution of the diagnostic characters of anterior caudal vertebrae in the Rebbachisauridae.

Taxa	Characters											
	1	2	3	4	5	6	7	8	9	10	11	12
*Amazonsaurus*	?	?	?	0	?	1	1	1	0	1	0	0
*Cathartesaura*	?	0	1	1	2	0	?	1	0	1	0	0
*Comahuesaurus*	1	0	0	1	2	1	0	0	0	?	?	0
*Demandasaurus*	1	0	1	1	2	1	0	0	1	0	1	1
*Dzharatitanis*	1	0	1	1	1	1	1	1	0	0	1	1
*Itapeusaurus*	?	0	1	1	2	0	?	0	0	0	1	0
*Katepensaurus*	?	?	1	1	1	0	?	1	0	1	0	1
*Lavocatisaurus*	?	1	?	1	?	?	?	?	0	?	?	0
*Limaysaurus*	0	1	?	1	?	?	?	0	0	1	0	0
*Nigersaurus*	?	0	?	?	?	1	0	0	0	1	0	1
*Rebbachisaurus*	?	?	?	?	?	?	?	?	?	0	0	1
*Tataouinea*	0	1	?	1	2	0	?	0	1	1	0	1
Bajo Barreal taxon [47]	?	1	1	1	0	0	?	0	0	1	0	0
Kem Kem taxon [48]	?	1	1	1	0	1	0	?	1	?	?	?
Wessex taxon [49]	?	?	1	1	2	1	0	0	1	1	0	1

Characters: 1, first caudal, centrum anterior articular surface (0, concave; 1, flat or convex); 2, centrum, pleurocoel (0, absent; 1, present); 3, transverse process, shape (0, triangular, tapering distally; 1, wing-like); 4, transverse process, ventral surface directed dorsally (0, absent; 1, present); 5, PRCDF (0, small; 1, large, shallow; 2, large, deep); 6, TPRL (0, present; 1, absent); 7, prezygapophyses ventral contact (only taxa without TPRL) (0, present; 1, absent); 8, postzygapophyses ventral contact (0, present; 1, absent); 9, hyposphenal ridge (0, absent; 1, present); 10, SPRL and SPOL contact (lateral lamina) (0, absent; 1, present); 11, SPDL (0, absent; 1, present); 12, triangular lateral process (0, absent; 1, present).

The anterior articular surface of the centrum in *Dzharatitanis* is slightly convex. This could be a positional feature, as the holotypic vertebra of *D*. *kingi* is likely a first caudal. Among rebbachisaurids, a slightly convex or flat anterior articular surface of the first caudal centrum is present in *Comahuesaurus* [33] and *Demandasaurus* [27]. The anterior centrum articular surface of the first caudal is concave in *Limaysaurus* [44] and *Tatouinea* [36]. The first caudal of *D*. *kingi* with its slightly convex anterior and more deeply concave posterior articular surfaces is technically opisthocoelous (character 190(2)). Opisthocoelous centra of anterior caudals represent a rare character state among sauropods. Strongly opisthocoelous anterior caudals are currently known only in two Late Cretaceous sauropod taxa–*Opisthocoelicaudia skarzynskii* from the Campanian-Maastrichtian Nemegt Formation of Mongolia [50] and *Abdarainurus barsboldi* from the Campanian(?) Alagteeg Formation of Mongolia [51]. Another sauropod with opisthocoelous anterior caudals is the titanosauriform *Daxiatitan binglingi* from the Aptian Hekou Group of Gansu, China [52]. Originally the anterior caudal of the latter taxon was interpreted as procoelous, but a well preserved postzygapophysis on the concave side of the centrum [52] indicates that the vertebra is opisthocoelous.

The centrum of USNM 538127 lacks pleurocoels (character 194(0)). All taxa included in the data matrix were coded initially as lacking pleurocoels, which is incorrect for *Limaysaurus* [44], for which the coding has accordingly been changed (0→1). We also changed the coding for *Cathartesaurua* (?→0) [46]. The absence of the pleurocoel on the anterior caudal centra is also documented for *Comahuesaurus* [33], *Demandasaurus* [26, 27], *Itapeusaurus* [41], and *Nigersaurus* [13]. Among rebbachisaurids other than *Limaysaurus*, the pleurocoels on the anterior caudal centra are present in *Lavocatisaurus* [35], *Tataouinea* [36], the Bajo Barreal rebbachisaurid [47], and the Kem Kem rebbachisaurid [48].

The transverse process of USNM 538127 is dorsoventrally expanded and ‘wing-like’ (character 198(1)). This character-state is considered a synapomorphy of Flagellicaudata ([53]; this analysis) or for *Amphicoelias* + more derived diplodocoids [54]. Among rebbachisaurids other than *Dzharatitanis*, ‘wing-like’ transverse processes on anterior caudals are present in *Cathartesaura* [46], *Demandasaurus* [27] [coding of this taxon changed in the matrix: 0→1], *Itapeusaurus* [41], *Katepensaurus* [45], the Bajo Barreal rebbachisaurid [47], the Kem Kem rebbachisaurid [48], and the Wessex rebbachisaurid [49]. The transverse processes of the anterior caudals are triangular and tapering distally in *Comahuesaurus* [33]. The coding of this character has also been changed in the matrix for *Limaysaurus* (0→?).

The ventral side of the transverse process in USNM 538127 is directed dorsolaterally (character 192(1)). This character is considered a synapomorphy of Rebbachisauridae in the analyses by Canudo et al. [35] and in this paper. In the analysis of Whitlock [54], this character was hypothesized as a synapomorphy of *Amargasaurus* + more derived dicraeosaurids and Rebbachisaurinae (= “Limaysaurinae”) + Nigersaurinae. Among rebbachisaurids, the transverse processes on the anterior caudal vertebrae are directed laterally only in *Amazonsaurus* [32]. In other taxa, the ventral margin of the transverse process is dorsolaterally directed: *Comahuesaurus* [33], *Demandasaurus* [27], *Itapeusaurus* [41], *Katepensaurus* [45], *Lavocatisaurus* [35], *Limaysaurus* [44], *Tataouinea* [36], the Bajo Barreal rebbachisaurid [47], the Kem Kem rebbachisaurid [48], and the Wessex rebbachisaurid [49].

The anterior side of the transverse process in USNM 538127 is excavated by the PRCDF. The PRCDF is present in all rebbachisaurids but varies in structure. The PRCDF is small in the Bajo Barreal rebbachisaurid [47] and the Kem Kem rebbachisaurid [48]. In *Dzharatitanis* (Fig 3A and 3B) and *Katepensaurus* [45], the PRCDF is large but shallow and subdivided by an oblique ridge. The other rebbachisaurid taxa have a derived condition of the PRCDF where the fossa forms a deep excavation between the thin posterior wall of the transverse process and the thick dorsal and ventral bony bars; the latter bar forms a horizontal plate. This derived feature is present in *Cathartesaura* [46], *Comahuesaurus* [33], *Demandasaurus* [27], *Itapeusaurus* [41], and the Wessex rebbachisaurid [49]. The transverse processes of the anterior caudals of *Tataouinea* are incompletely preserved, but the dorsal portion of the transverse process on the fifth caudal [36] suggests that this taxon shares the derived condition. In *Itapeusaurus* and the Wessex rebbachisaurid, the PRCDF is also subdivided by a subvertical or subhorizontal ridge.

An unusual character of USNM 538127 is the absence of transverse laminae connecting the prezygapophyses and postzygapophysis–the TPRL and TPOL, respectively. The TPRL, a horizontal plate between the separated ventral margins of the prezygapophyses, is present on anterior caudal vertebrae in Dicraeosauridae, Diplodocidae, *Katepensaurus* [45], *Tataouinea* [36], the Bajo Barreal rebbachisaurid [47], and possible in *Cathartesaura* [46]. In *Itapeusaurus*, the TPRL is present on the first caudal vertebra but absent on a more distal anterior caudal vertebra [41]. The TPRL is absent but the prezygapophyses contact each other ventrally in *Comahuesaurus* [33], *Demandasaurus* [27], *Nigersaurus* [13], the Kem Kem rebbachisaurid [48], and the Wessex rebbachisaurid [49]. The TPRL is absent, the ventral margins of prezygapophyses are separated, and the PRSL extends to the roof of the neural canal in *Dzharatitanis* and *Amazonsaurus* [32].

The TPOL, as a horizontal lamina between the separated ventral margins of the postzygapophyses on anterior caudal vertebrae, is absent in all known rebbachisaurids. The postzygapophyses contact each other ventrally on the anterior caudals in *Comahuesaurus* [33], *Demandasaurus* [27], *Itapeusaurus* [41], *Limaysaurus* [44], *Nigersaurus* [13], *Tataouinea* [36], the Bajo Barreal rebbachisaurid [47], and the Wessex rebbachisaurid [49]. The postzygapophyses are separated ventrally in *Dzharatitanis*, *Amazonsaurus* [32], *Cathartesaura* [46], and *Katepensaurus* [45].

USNM 538127 lacks a hyposphenal ridge (character 203(0)). This character state is considered a synapomorphy for Rebbachisauridae in previous analyses [35] and the analysis presented in this paper. The absence of the hyposphenal ridge has been confirmed for *Amazonsaurus* [32], *Cathartesaura* [46], *Comahuesaurus* [33], *Itapeusaurus* [41], *Katepensaurus* [45], *Lavocatisaurus* [35], *Limaysaurus* [44], *Nigersaurus* [13], and the Bajo Barreal rebbachisaurid [47]. However, among Rebbachisauridae, the hyposphenal ridge is present in *Demandasaurus* [26, 27], *Tataouinea* [36], the Wessex rebbachisaurid [49], and the Kem Kem rebbachisaurid [48].

USNM 538127 has a triangular lateral process on either side of the apex of its neural spine (character 197(1)). This character-state was considered a synapomorphy of Nigersaurinae [54]. Aside from *Dzharatitanis*, the triangular lateral process is present in *Demandasaurus* [26, 27], *Nigersaurus* [13], *Katepensaurus* [45], *Tataouinea* [36], *Rebbachisaurus* [40], and the Wessex rebbachisaurid [49]. In the character-taxon matrix used here, *Rebbachisaurus* was coded as unknown for this character, but the neural spine of the anterior caudal of the holotype of *R*. *garasbae* [40] has a profile of the lateral margin in anterior or posterior view with a lateral triangular projection almost identical to that in *Nigersaurus* [13]. Both taxa should be coded as having the same state for this character and accordingly we have changed the coding for *Rebbachisaurus* (?→1). The absence of a triangular lateral process on the neural spine among rebbachisaurids is documented for *Amazonsaurus* [32], *Itapeusaurus* [41], *Lavocatisaurus* [35], *Limaysaurus* [44], and the Bajo Barreal rebbachisaurid [47]. *Cathartesaura* and *Comahuesaurus* are also coded as lacking the triangular lateral process in the character-taxon matrix used in this paper. Outside Rebbachisauridae, a triangular lateral process is present in a dicraeosaurid *Lingwulong* from the Lower-Middle Jurassic (Toarcian–Bajocian) Yanan Formation of the Ningxia Hui Autonomous Region, China, but only on the first caudal vertebra [19].

In USNM 538127, the SPRL and SPOL do not contact each other on the lateral aspect of the neural spine and separately join the expanded distal end of the neural spine (character 206(0)). On the anterior caudal vertebrae of Diplodocidae, a composite lamina on the dorsal part of the lateral surface of the neural spine is formed by the fusion of the SPRL and SPOL [11, 53]. The presence of this composite lateral lamina was also considered a synapomorphy for Rebbachisaurinae (=“Limaysaurinae”) [54], which is confirmed by our analysis.

Among Rebbachisauridae, the contact of SPRL and SPOL on the lateral aspect of the neural spine is absent, aside from *Dzharatitanis*, in *Demandasaurus* [27], *Itapeusaurus*, *Rebbachisaurus*, and the Wessex rebbachisaurid (see discussion below). These laminae contact each other, forming a composite lateral lamina in *Amazonsaurus* [32] (coding corrected 0,1→1), *Cathartesaura* [46], *Katepensaurus* [45], *Limaysaurus* [44], *Nigersaurus* [13, 31], *Tataouinea* [36], and the Bajo Barreal rebbachisaurid [47].

*Dzharatitanis* is distinctive in having a strong SPDL on the lateral side of the neural spine, which is separated from SPRL and SPDL. Among rebbachisaurids, a similar condition is found only in *Demandasaurus* [27], *Itapuesaurus* [41] and Wessex rebbachisaurid [49]. In *Tataouinea* SPDL is well developed on the sacral vertebrae, and there is a short separate SPDL on the third caudal vertebra, but this lamina is absent on more posterior caudals [36]. In other rebbachisaurids, the lateral lamina of neural spine is formed by fusion of SPRL and SPOL (see above).

## Discussion

Nesov [55] referred long, ‘pencil-shaped’ sauropod teeth with smooth enamel from the Upper Cretaceous of Uzbekistan to either Diplodocidae or Titanosauridae. Subsequently, all sauropod remains from Dzharakuduk were referred to Titanosauridae [1] or Titanosauria [2–4]. The presence of the latter is still supported based on the sauropod braincase CCMGE 628/12457 [2, 7–10] by the phylogenetic analysis in this paper. Thus, two sauropod taxa can currently be recognized from the Bissekty Formation at Dzharakuduk, a rebbachisaurid, *Dzharatitanis kingi*, based on the anterior caudal USNM 538127, and an unidentified titanosaurian, based on the braincase CCMGE 628/12457. The identification of other fragmentary sauropod specimens from Dzharakuduk, including numerous isolated teeth, is more problematical.

All sauropod teeth from Dzharakuduk were previously referred to Titanosauria [2, 3]. However, the structure of some of these teeth is also consistent with that in Rebbachisauridae. Very similar isolated sauropod teeth from the Upper Cretaceous Bauru Group of Brazil, initially referred to Titanosauria [56], were reinterpreted by Sereno and Wilson [30] as belonging to Rebbachisauridae. Similar teeth are also known from the Lower Cretaceous (Barremian) Wessex Formation of the Isle of Wight, United Kingdom [57] and in *Nigersaurus* [30, 31]. However, an isolated rebbachisaurid tooth from the Barremian La Amarga Formation of Argentina, illustrated in fig. 3F-I in [58] is considerably expanded at mid-height, a condition not observed in any sauropod tooth in the sample from Dzharakuduk. In *Demandasaurus*, the tooth crowns have mesial and distal carinae and are ornamented with longitudinal crests on the labial and lingual sides [27]. Some sauropod teeth from Dzharakuduk bear carinae [3] but they are at most slightly expanded mesiodistally or have parallel distal and mesial margins, whereas the tooth crowns of *Demandasaurus* taper apically in labial or lingual view. The teeth of *Lavocatisaurus* [35] are similar to those from Dzharakuduk in shape but have prominent longitudinal crests, as in *Demandasaurus*. Most sauropod teeth from Dzharakuduk lack these crests but they are present on ZIN PH 2416/16 [3]. Rebbachisaurid teeth are also characterized by asymmetrical enamel thickness, being thicker on the labial side than on the lingual side [27, 30, 35], a condition also found on some sauropod teeth from Dzharakuduk. One of the notable characters of the sauropod teeth from Dzharakuduk is the reduction of the wrinkled enamel texture [3]. The tooth enamel is smooth is some titanosaurs but also in the rebbachisaurids *Demandasaurus* and *Limaysaurus* [27, 44].

A juvenile sauropod dorsal centrum USNM 538133 from Dzharakuduk, previously identified as titanosaurian [2], has a large pleurocoel extending for most of the length of the centrum. Comparably large pleurocoels on dorsal vertebrae are present in *Rebbachisaurus* [40].

Cylindrical middle caudal vertebrae, with flat ventral margins, were considered a synapomorphy for Rebbachisauridae [33, 40]. However, we were not able to find any illustrations of rebbachisaurid middle caudals that show this feature. Furthermore, the middle caudal vertebrae in *Tataouinea* have centra with deeply concave ventral margins in lateral view, with variously developed grooves and foramina [36]. The middle-posterior caudal vertebra ZIN PH 962/16 from Dzharakuduk [2] is comparable in proportions to the middle caudal of *Limaysaurus* [44]. Its centrum has a similarly concave ventral margin in lateral view, but the vertebra differs in having a lower neural spine, which is subject to positional variation. ZIN PH 962/16 is also similar to the posterior caudal vertebra of *Amazonsaurus* [32] in the bending of the centrum. The centrum of ZIN PH 962/16 is amphicoelous, as in rebbachisaurids. Alternatively, this specimen could belong to a non-lithostrotian titanosaurian. The middle-posterior caudal vertebra of *Demandasaurus* is distinctive in having longitudinal crests on the lateral surface of the centrum [27]. There are no such crests on ZIN PH 962/16.

Among Rebbachisauridae, *Amazonsaurus*, *Katepensaurus*, and *Tataouinea* show a derived level of postcranial pneumaticity [36, 37, 59]. USNM 538133 shows remarkable pneumaticity of its neural arch and transverse process but the centrum is solid and lacks pleurocoels, in contrast with *Tataouinea*, which has pneumaticized anterior caudal centra [36]. The neural arch and transverse process of USNM 538133 have prominent fossae that likely housed pneumatic diverticula that originated from the abdominal air sacs [59–61] and include PRCDF and POCDF on the anterior and posterior sides of the transverse process, respectively; POSDF on the lateral aspect of the neural spine; and PRSDF and SPOF on the anterior and posterior sides of the neural spine, respectively (Fig 3). The fossae in the neural spine contain small pneumatic foramina that open into the internal cavity of the neural spine. USNM 538133 resembles the anterior caudals of *Katepensaurus* in having the pneumatic foramina within the SPRF, posterior to the prezygapophyseal articular surface [45], and a shallow PRCDF. Some other rebbachisaurids have hypertrophied PRCDF that undoubtedly housed a large pneumatic diverticulum (see above). The pneumatic features in USNM 538133 are asymmetrical, being developed to a greater extent on the left side, as is the case in *Tataouinea* [36].

Ibiricu et al. [59] interpreted increases in postcranial pneumaticity in rebbachisaurids as an adaptation for living under extraordinarily high ambient temperatures. However, only two of four known rebbachisaurid taxa with increased postcranial pneumaticity (*Katepensaurus* and *Dzharatitanis*) existed during the Cretaceous thermal maximum [59]. Furthermore, rebbachisaurids had a wide palaeolatitudinal distribution and thus were likely able to adapt to a variety of climatic conditions.

The Rebbachisauridae are interesting from the palaeobiogeographic point of view because they were mainly present in Africa and South America, supporting the hypothesis that these continents were still connected during the Early Cretaceous [62, 63]. The differentiation of rebbachisaurids into Afro-European and South American clades has been interpreted as the result of a vicariance event following the separation of Africa and South America by the emerging South Atlantic Ocean [31, 43, 54]. However, there is no current consensus regarding the ingroup relationships of Rebbachisauridae (Table 3). The stratigraphically oldest and basalmost rebbachisaurid is the Hauterivian-Barremian *Histriasaurus*, which differs from more derived rebbachisaurids in the presence of a triangular hyposphene on the dorsal vertebrae [33, 58]. It comes from the Apulian Plate, which is now part of Europe (present-day Croatia) but was part of Africa during the Early Cretaceous [25, 27]. The presence of the rebbachisaurid *Demandasaurus* on the Iberian Plate in the Barremian-Aptian of Europe (present-day Spain) has been explained as a dispersal from Africa [26, 64]. Torcida Fernández-Baldor et al. [27] proposed an alternative explanation of this occurrence as the result of vicariance. This explanation is implausible because Rebbachisauridae did not exist when Europe and Africa were still united within the supercontinent Pangea. The Iberian plate has been separated from Africa since the Early Jurassic [65, 66] and the date for the origin of Diplodocoidea has been estimated as early Middle Jurassic [19]. Dispersal of rebbachisaurids from Africa to Europe was possible at this time via the Apulian Route [27, 33, 41, 43, 64]. *Histriasaurus* does not represent an example of dispersal by a mechanism that has been termed “Noah’s Ark” ([67]; contra Torcida Fernández-Baldor et al. [27]) because the Apulian Plate was still part of Africa, not Europe, during the Early Cretaceous [68]. Thus, the occurrence of *Histriasaurus* cannot be considered the result of a dispersal event [36].

**Table 3:** Phylogenetic content of the subgroups within the Rebbachisauridae in previous studies.

Rebbachisaurinae	Nigersaurinae	Limaysaurinae	References
	*Nigersaurus*, *Demandasaurus*, *Zapalasaurus*	*Limaysaurus*, *Cathartesaura*	[54]
*Rebbachisaurus*, *Rayososaurus*, *Cathartesaura*, *Limaysaurus*	*Nigersaurus*, *Demandasaurus*		[33]
*Rebbachisaurus*, *Rayosasaurus*, *Cathartesaura*, *Limaysaurus*	*Nigersaurus*, *Demandasaurus*, *Tataouinea*		[37]
*Katepensaurus*, *Nigersaurus*, *Rebbachisaurus*, *Demandasaurus*, *Tataouinea*		*Limaysaurus*, *Cathartesaura*	[35, 36]
	*Nigersaurus*, *Demandasaurus*	*Limaysaurus*, *Cathartesaura*, *Katepensaurus*	[69]
*Rebbachisaurus*, *Nigersaurus*, *Demandasaurus*		*Limaysaurus*, *Cathartesaura*	[40]
	*Nigersaurus*, *Demandasaurus*, *Itapeusaurus*	*Limaysaurus*, *Cathartesaura*	[41]
*Nigersaurus*, *Rebbachisaurus*, *Tataouinea*, *Demandasaurus*		*Nopcsaspondylus*, *Rayososaurus*, *Cathartesaura*, *Limaysaurus*	[70]

It is most likely that the rebbachisaurids dispersed to Central Asia from Europe but it is not clear when this could have occurred. Rare isolated teeth from the Cenomanian localities of Central Asia do not show prominent longitudinal crests or carinae and likely are all referable to Titanosauria [4]. For most of the Cretaceous Period, Asia was separated from Europe by the Turgai Strait, but a land connection between the two landmasses existed during the early Berriasian, late Valanginian, early Hauterivian, Barremian, late Aptian, late Albian, and Cenomanian [71]. The Rebbachisauridae were present in Europe at least during the Barremian (*Demandasaurus*, Wessex rebbachisaurid). *Dzharatitanis* shares with these two European rebbachisaurids at least one unique feature, a strong SPDL on lateral side of the neural spine, which is separate from SPRL and SPOL. These three taxa may form a natural group. The dispersal of rebbachisaurids from Europe to Asia could have occurred at any time during the interval from the Barremian to the Turonian.

As noted by Wilson and Allain [40], the Rebbachisauridae have a restricted palaeolongitudinal and expanded palaeolatidunal distribution. All previous records of rebbachisaurids come from a narrow band extending from southernmost South America through the northeastern South America and northwestern Africa to Europe. The discovery of the first Asiatic rebbachisaurid, *Dzharatitanis kingi*, now considerably extends the known distribution of the group to the east.

## Conclusions

An isolated sauropod anterior caudal vertebra USNM 538127, most likely a first caudal, from the Upper Cretaceous (Turonian) Bissekty Formation at Dzharakuduk in the Kyzylkum Desert of Uzbekistan, was referred previously to an indeterminate titanosaurian but is here reinterpreted as the first Asiatic record of the diplodocoid clade Rebbachisauridae. The caudal is characterized by complex lamination and intensive pneumatization of the neural arch. Its centrum is slightly opisthocoelous, as in some rebbachisaurids. The transverse process is ‘wing-like’, with large but shallow PRCDF anteriorly and POCDF posteriorly, both of which are subdivided by ridges. There are no transverse laminae connecting the pre- and postzygapophyses, respectively (TPRL and TPOL). The postzygapophyses do not contact each other ventrally. A hyposphenal ridge is absent. The SPRL and SPOL do not contact each other on the lateral aspect of the neural spine and extend in parallel towards the apex of the spine. Between these laminae there is a prominent separate SPDL. The neural spine is hollow. Its apex is transversely expanded and bears triangular lateral processes. By the combination of these diagnostic features, USNM 538127 can be clearly distinguished from all other known rebbachisaurid taxa and is designated here as the holotype of a new taxon, *Dzharatitanis kingi* gen. et sp. nov. The prominent SPDL separate from SPRL and SPOL unites *Dzharattanis* with Europaean rebbachisaurids (*Demandasaurus* and Wessex rebbachisaurid). *Dzharatitanis kingi* is the first rebbachisaurid reported from Asia and one of the geologically youngest known records of this clade. The Rebbachisauridae possibly dispersed from Europe to Asia via a land bridge across the Turgai Strait sometime between the Barremian and Turonian. The sauropods from the Bissekty Formation now comprise at least two taxa, the rebbachisaurid *Dzharatitanis kingi* and an indeterminate titanosaurian.

## Supporting information

S1 File Scorings for the matrix of Rauhut et al., 2015, modified by Xu et al., 2018. (DOCX)

S2 File (TNT)
