# Supplementary material for: First rebbachisaurid sauropod dinosaur from Asia
Source: PLoS One. 2021 Feb 24;16(2):e0246620. doi: 10.1371/journal.pone.0246620 (PMC7904184; doi:10.1371/journal.pone.0246620)
Supplement: S1 File — (DOCX) [file pone.0246620.s001.docx]

**S1 File. Scorings for the matrix of Rauhut et al., 2015, modified by Xu et al., 2018.**

USNM 538127, anterior caudal vertebra, the holotype of *Dzharatitanis kingi* gen. et sp. nov.

188(0). Caudal bone texture: solid.

190(2). First caudal centrum or last sacral vertebra, articular face shape: opisthocoelous.

191(1). First caudal neural arch, coel on lateral aspect of neural spine: present.

192(1). Anterior caudal vertebrae, transverse processes: ventral surface directed dorsally.

194(0). Anterior caudal centra, pleurocoels: absent.

195(0). Anterior caudal vertebrae, ventral surfaces: convex transversely.

196(0). Anterior and middle caudal vertebrae, ventrolateral ridges: absent.

197(1). Anterior and middle caudal vertebrae, triangular lateral process on the neural spine: present.

198(1). Anterior caudal transverse processes shape: "wing-like", not tapering distally.

199(1). Anterior caudal neural spines, transverse breadth: greater than anteroposterior length.

200(1). Anterior caudal transverse processes, proximal depth: deep, extending from centrum to neural arch.

201(0). Anterior caudal transverse processes, diapophyseal laminae (ACDL, PCDL, PRDL, PODL): absent.

203(0). Anterior caudal vertebrae, hyposphene ridge: absent.

205(0). Anterior caudal neural arches, spinoprezygapophyseal lamina (SPRL): absent, or present as small short ridges that rapidly fade out into the anterolateral margin of the spine.

206(0). Anterior caudal neural arches, spinoprezygapophyseal lamina (SPRL)-spinopostzygapophyseal lamina (SPOL) contact: absent.

207(1). Anterior caudal neural arches, prespinal lamina (PRSL): present.

209(0). Anterior and middle caudal centra, ventral longitudinal hollow: absent.

Braincase CCMGE 628/12457

40(0). Parietal occipital process, dorsoventral height: short, less than the diameter of the foramen magnum.

41(0). Parietal, contribution to post-temporal fenestra.

50(0). Supraoccipital, sagital nuchal crest: broad, weakly developed.

65(1). Supraoccipital, height: twice subequal to (0); or less than height of foramen magnum.

67(0). Crista prootica, size: rudimentary.

69(0). Basipterygoid processes, angle of divergence: approximately 45°.

70(0). Basal tubera, anteroposterior depth: approximately half dorsoventral height.

72(0). Basal tubera: distinct from basipterygoid.

73(0). Basal tubera, shape of posterior face: convex.

74(1). Basioccipital depression between foramen magnum and basal tubera: present.

75(0). Basisphenoid/basipterygoid recess: present.

77(0). Basisphenoid, sagital ridge between basipterygoid processes: absent.

79(0). Basipterygoid, area between the basipterygoid processes and parasphenoid rostrum: is a mildly concave subtriangular region.

80(1). Occipital region of skull, shape: flat, paroccipital processes oriented transversely.

372(0). Exoccipital – small, deep, horizontally oriented groove immediately lateral to each of the proatlantal facets: absent.

Changes to the data matrix (see discussion in the text)

*Amazonsaurus*: 206(0,1→1)

*Cathartesaura*: 194(?→0)

*Demandasaurus*: 198(0→1)

*Limaysaurus*: 194 (0→1), 198 (0→1)

*Rebbachisaurus*: 197(?→1), 206(?→0)
